# Curcumin Nanoparticle Enhances the Anticancer Effect of Cisplatin by Inhibiting PI3K/AKT and JAK/STAT3 Pathway in Rat Ovarian Carcinoma Induced by DMBA

**DOI:** 10.3389/fphar.2020.603235

**Published:** 2021-01-18

**Authors:** Ni Made Dwi Sandhiutami, Wawaimuli Arozal, Melva Louisa, Deni Rahmat, Puspita Eka Wuyung

**Affiliations:** ^1^ Doctoral Program in Biomedical Sciences, Faculty of Medicine, Universitas Indonesia, Jakarta, Indonesia; ^2^ Faculty of Pharmacy, University of Pancasila, Jakarta, Indonesia; ^3^ Department of Pharmacology and Therapeutics, Faculty of Medicine, Universitas Indonesia, Jakarta, Indonesia; ^4^ Department of Pathological Anatomy, Faculty of Medicine, Universitas Indonesia, Jakarta, Indonesia; ^5^ Animal Research Facility, Indonesian Medical Education and Research Institute, Jakarta, Indonesia

**Keywords:** ovarian carcinoma, cisplatin, curcumin, nanoparticles, TGF-β, interleukin-6

## Abstract

Cisplatin has been used for decades for the treatment of ovarian cancer. However, despite its potent anticancer effect, cisplatin’s efficacy as a single agent was inadequate in patients with advanced stage. Curcumin has been shown to sensitize cisplatin activity in several cancer models. However, the low bioavailability of curcumin has limited its anticancer potential. Hence, nano-formulation of curcumin was developed to increase its therapeutic efficacy in ovarian cancer. The objective of this study was to investigate the mechanism of curcumin nanoparticles given in combination with cisplatin in rat ovarian carcinoma induced by dimethylbenz(a)anthracene (DMBA). The administration of cisplatin and nanocurcumin resulted in a significant reduction in ovarian tumor volume and weight. Furthermore, there were reduction in expressions of Ki67, TGF-β, PI3K, and Akt phosphorylation. Co-treatment of cisplatin and nanocurcumin also reduced JAK expression, STAT3 phosphorylation, and reduced IL-6 concentrations. Altogether, nanocurcumin, given as a co-treatment with cisplatin has therapeutic potential in ovarian cancer models by inhibiting proliferation through downregulation of PI3K/Akt and JAK/STAT3 signaling pathways.

## Introduction

Ovarian cancer is the leading cause of mortality and is currently one of the five most common women cancers globally. It is estimated that by the year of 2040, cancer mortality rates will increase significantly (Bray et al., 2018). In the case of ovarian cancer, the high cancer mortality rate is caused due to asymptomatic symptoms, delayed onset of symptoms, late diagnosis, and lack of proper screening. All of these factors result in tendency for ovarian cancer to be diagnosed at an advanced stage with four out of five ovarian cancer patients are diagnosed at an advanced stage. When the ovarian cancer has metastasized to the abdomen, the 5-years-survival rate is only 29% (Allemani et al., 2015). The therapeutic modality for ovarian epithelial cancer is cytoreduction surgery followed by chemotherapy. The most common chemotherapy used is platinum (cisplatin or carboplatin). However, the effectiveness of chemotherapy is only about 60–80% and cancer recurrence are common (Markman, 2019; Mikuła-Pietrasik et al., 2019).

Previous studies showed hyperactivation of the phosphoinositol-3-kinases (PI3K), AKT, mTOR (PI3K/AKT) pathway in nearly 60% of patients with ovarian cancer. This pathway plays a significant role in cancer cell growth, cell survival, programming of metabolic, autophagy, regulation of transcription, and angiogenesis (Ghoneum and Said, 2019; Huang et al., 2020b). Activation of the PI3K pathway causes downstream Protein Kinase B (PKB) signaling activation. This protein will then phosphorylate other intracellular proteins with subsequent activation of cell cycle regulation, cell proliferation, DNA repair systems, and apoptosis (Akinleye et al., 2013). Cisplatin resistance is associated with altered activation of signaling pathways, including PI3K/Akt/mTOR. Some signaling pathway components include PI3K and AKT represents potential therapeutic targets (Zhang et al., 2016; Gohr et al., 2017).

Cytokine receptor signals transduction through the JAK-STAT pathway also causes chemotherapy resistance through apoptosis inhibition in epithelial cancer including ovarian cancer. STAT3 activation is accommodated by Janus kinase protein. STAT3 is activated in various types of tumors, and this activation can accelerate cell proliferation, increase regulation of cell survival factors, and activate anti-apoptotic markers (Bharadwaj et al., 2020). Currently, STAT3, an oncogenic transcription factor, is a potential pharmacological target. Several preclinical studies have demonstrated that inhibiting STAT3 activation resulted in reduced cell growth and increased apoptosis in tumor cells (Kuppusamy et al., 2008; Selvendiran et al., 2008; Yu et al., 2009; Bose et al., 2020).

The addition of standard chemotherapy in ovarian cancer treatment with agents that work distinctively to the signaling pathway target can increase chemotherapy’s potential and increase the survival rate in advanced ovarian cancer. Several natural compounds, including curcumin, have been widely tested as adjunctive treatment to the available anticancer (Qin et al., 2017; Khan et al., 2018). Curcumin has been well documented to have a wide range of pharmacological effects, including antimicrobial, anti-inflammatory, antioxidant, anti-diabetic, wound healing activities, and anti-osteoporotic (Hussain et al., 2017; Dong et al., 2018; Hussain et al., 2020). As an anticancer, curcumin was shown to inhibit tumor growth, angiogenesis and induce apoptosis. Curcumin worked on multiple steps of carcinogenesis. It is established that curcumin work by inhibiting tumor growth, angiogenesis, and inducing apoptosis (Park et al., 2013; Montané et al., 2020; Thyagarajan et al., 2020).

The combination of cisplatin and curcumin has been used in many studies both *in vitro* and *in vivo*. While numerous *in vitro* studies has been done, *in vivo* study in animal cancer model is not as prevalent (Duarte et al., 2010; Bortel et al., 2015; Fetoni et al., 2015; Kumar et al., 2017; Gökçe Kütük et al., 2019). In the breast cancer animal model, curcumin potentiates cisplatin antitumor activity by improving several inflammatory markers, increasing the expression of PPAR-γ, and decreasing BDNF expressions (Kumar et al., 2017). Curcumin was shown to re-sensitize cisplatin’s effect by targeting pSTAT3 and Nrf2 in head and neck cancer (Fetoni et al., 2015). In hepatocellular carcinoma, curcumin enhances cisplatin’s antitumor activity by inhibiting NF-kB, β-catenin, and decreasing cyclin D (Bortel et al., 2015).

Despite these anti-cancer mechanisms, the potent anticancer activity of curcumin is limited by its poor bioavailability. Oral administration of curcumin only produce low levels of blood curcumin concentration due to several factors such as low water solubility and high first-pass metabolism (Heger et al., 2014). To date, nanotechnology has been widely utilized in many applications in cancer research, including early detection technology, prognosis determination, and treatment strategy (Choudhury et al., 2018; Gao et al., 2018; Hussain et al., 2018; Safdar et al., 2018). Several formulations of nanoparticle curcumin have been developed to improve its bioavailability and inhibit its rapid metabolic process of curcumin (Karthikeyan et al., 2020). Curcumin nano-formulations have allowed their applications in many therapeutic conditions, including cancer (Yadav et al., 2012; Yallapu et al., 2012; Wong et al., 2019).

We have recently developed a formulation of curcumin loaded chitosan nanoparticles using ionic gelation methods (Arozal et al., 2020). Our previous study has shown that our nanocurcumin formulation has an improved pharmacokinetic profile compared with traditional curcumin, with a 16 times increase in AUC_0-∞_ (Arozal et al., 2020). In the present study, our formulation of curcumin nanoparticles was tested as co-treatment with cisplatin in a rat ovarian cancer model, with a focus on PI3K/Akt and JAK/STAT3 pathways.

## Material and Methods

### Nanocurcumin Formulation

The formulation of nanocurcumin was carried out at the Faculty of Pharmacy, University of Pancasila. Nanocurcumin was made by loading curcumin to chitosan-sodium tripolyphosphate using the method previously described (Arozal et al., 2020). Curcumin was obtained from Plamed Green Science Limited, Xian, China (total curcuminoid content of ≥95%). The particle size of nanocurcumin used in this study was 11.5–30.6 nm, while the particle size of curcumin was about 15,000–16,000 nm. The full characterization of the nanocurcumin used in this study, including the transmission electron microscope (TEM) image, polydispersity index, zeta potential, entrapment efficiency, mucoadhesive properties, and stability, were described in the previous manuscript (Arozal et al., 2020).

### Animals and Treatments

This study was approved by the Health Research Ethics Committee’s Faculty of Medicine, Universitas Indonesia. Our experiment was conducted on 25 female Wistar rats. Dimethylbenz-(a)anthracene (DMBA)-induced ovarian cancer was conducted on 20 rats. We implanted silk coated with 7,12-dimethylbenz(a)anthracene (DMBA) on the ovary of the rats using the method previously described (Dwi Sandhiutami et al., 2019). We did sham implantation (silk with no DMBA coating) on the other five rats. It took at least 28 weeks from the implantation process to form a tumor period. After 28 weeks, the 20 DMBA-induced rats, were divided into four groups of treatments, each consists of five rats: vehicle only (OC); cisplatin group (4 mg/kg BW every week) (OC + Cis); cisplatin (4 mg/kg BW every week) with unmodified curcumin (100 mg/kg BW every day) (OC + Cis + Cur); cisplatin (4 mg/kg BW every week) with nanocurcumin (100 mg/kg BW every day) (OC + Cis + NC). Treatments were administered for four weeks. Afterward, the rats were euthanized and the ovaries were taken. The specimens then underwent gross pathology examination. Ovarian tissues were immediately dissected, weighted, and then stored at −80°C. Samples for histopathological examination were fixed in formalin solution for 12 h.

### Histopathological Preparation

After fixation in a 10% formalin solution overnight (12 h), tissue sections were carefully dissected from representative areas following the guidelines of the grossing technique given by standard books and processed in the automated tissue. Four μ thick sections of the representative paraffin-embedded blocks were cut using a rotatory microtome. The sections were then processed with hematoxylin and eosin (HE) and Ki67 immunohistochemical staining for histopathological examination and immunohistochemistry (IHC) study. The sections stained with routine HE staining were viewed under a light microscope and histopathological diagnosis was made based on the WHO classification of SEOT (Malpica et al., 2004).

Ki67 antigen immunostaining was carried out by standard immunohistochemistry method and peroxidase-anti peroxidase method using a monoclonal rabbit Ki67 antibody kit (SP6, Abcam) dan Histofine^®^ Simple stain™ MAX PO (Nichirei). A reactive tonsil was taken as a positive control. Ki-67 immunopositivity was observed as brown granular nuclear staining. For Ki67 scoring, the most immune-positive area of the tumor was selected, avoiding foci of inflammation. The number of immune-positive nuclei is counted in 1,000 tumor cells in at least 10 High Power Field (×40) under a binocular light microscope (Leica DM500) (Prat, 2015).

### Protein Isolation

Isolation of proteins from ovarian tissue using 10x RIPA buffer (50 mM Tris-HCl, pH 7.4, 1% NP-40, 0.25% Na-deoxycholate, 150 mM NaCl, 1 mM Na3VO4, and 1 mM NaF). Protein content was measured using Coomassie Plus (Bradford) assay kit using the Varioskan LUX Multimode Microplate Reader (Thermo Scientific) Spectrophotometer at a wavelength of 595 nm. The protein isolate was stored at –80°C for further analysis.

### Enzyme-Linked Immunoassay (ELISA)

Examination of TGF-β and IL-6 levels was carried out using a sandwich enzyme-linked immunosorbent assay method using TGF-β1 (Invitrogen, USA) and IL-6 (Invitrogen, USA) ELISA kit. The analysis was done following the manufacturer's protocol. The addition of a stop solution terminates the reaction, and absorbance is measured at 450 nm using Varioskan LUX Multimode Microplate Reader (Thermo Scientific) Spectrophotometer.

### Western Blot Analysis

PI3K, Akt and *p*-Akt, JAK, STAT3, and pSTAT3 protein expressions were measured using western blot (WB). All antibodies for Western blot analysis were purchased from Cell Signaling Technology (Beverly, MA). β-actin (13E5) Rabbit mAb (CST#4970), PI3 kinase p85 (19H8) Rabbit mAb (CST#4257), Akt antibody (CST#9272), *p*-Akt antibody (Ser473) (D9E) XP^®^ Rabbit mAb (CST#4060), JAK3 (D7B12) Rabbit mAb (CST#8863), STAT3 (D3Z2G) Rabbit mAb (CST#12640), phospho-Stat3 (Tyr705) (D3A7) XP^®^ Rabbit mAb (CST#9145), Anti Rabbit IgG HRP Link-Antibody. The sample used in testing was 70 μg of protein. SDS-page electrophoresis was done using a buffer tank Electrophoresis Apparatus Vertical TV100Y (Scie-Plas). Proteins that have been successfully separated will be transferred using semi-dry Trans Blot-SD Cell (Bio-Rad). The next stage is blocking by using skim milk and BSA. After blocking, the membrane is incubated with primary antibodies in a ratio of 1: 1,000. Incubation was carried out in Cold Room 4°C (Fiocetti) for 16–18 h. After incubation with a primary antibody, the membrane is washed and then incubated with secondary antibodies 1: 5,000. The target protein band on the membrane was detected using the Gel Documentation System with Chemiluminescence Alliance 4.7 (Uvitec) with enhanced chemiluminescence (ECL) substrate, Clarity Western.

### Quantitative RT-PCR Analysis

RNA was isolated from 20 mg of ovarian organ tissue using the Quick-RNA MiniPrepPlus kit (Zymo Research, CA, United States) and then synthesized to cDNA using ReverTra Ace^®^ qPCR RT Master Mix (Toyobo BioTech, Osaka, Japan) following the manufacturer’s protocols. The mRNA expressions of Bax, Bcl2, caspase-9, and caspase-3 were analyzed using qRT-PCR with β-actin as a reference gene. The primer sequences used for Bax, Bcl2, caspase-9, and caspase-3 are as follows: Bax Fwd: 5′-GAT​GCG​TCC​ACC​AAG​AAG​CT-3’; Bax Rev: 5′-CGG​CCC​CAG​TTG​AAG​TTG-3’ (Dai et al., 2017); Bcl-2 Fwd: 5′-GAG​CGT​CAA​CAG​GGA​GAT​G-3’; Bcl-2 Rev: 5′-GGA​TCC​AGG​TGT​GCA​GAT​G-3’; Caspase-3 Fwd: 5′-CTG​ACT​GGA​AAG​CCG​AAA​CT-3’; Caspase-3 Rev: 5′-GTT​CCA​CTG​TCT​GTC​TCA​ATA​CC-3’; Caspase-9 Fwd:5′-CCACTGCCTCATCATCAACA-3’; caspase-9 Rev: 5′-GTT​CTT​CAC​CTC​CAC​CAT​GAA-3’; β-actin Fwd: 5′-AGG​CCA​ACC​GTG​AAA​AGA​TG-3’; β-actin Rev: 5′-ACC​AGA​GGC​ATA​CAG​GGA​CAA-3’. The level of mRNA expressions was calculated using the Livak method (Livak and Schmittgen, 2001).

### Data Analysis

Data were presented as the mean ± standard deviation (SD). The homogeneity test (Levene test) with a significance value of homogeneity *p* > 0.05. A comparison of the groups' variable distribution was carried out using the One-Way ANOVA with a significance limit (*α*) 0.05. The analysis was continued by the least significant difference test (Tukey test). Pearson correlation was used to analyze relationships between two variables. All statistical analyzes were performed with SPSS version 26. All of the Graphs were presented in GraphPad Prism version 9.0.0.

## Results

### Improvement of Ovarian Macroscopic and Microscopic Features in Cisplatin–Curcumin/Nanocurcumin Treated Rats

**FIGURE 1:**
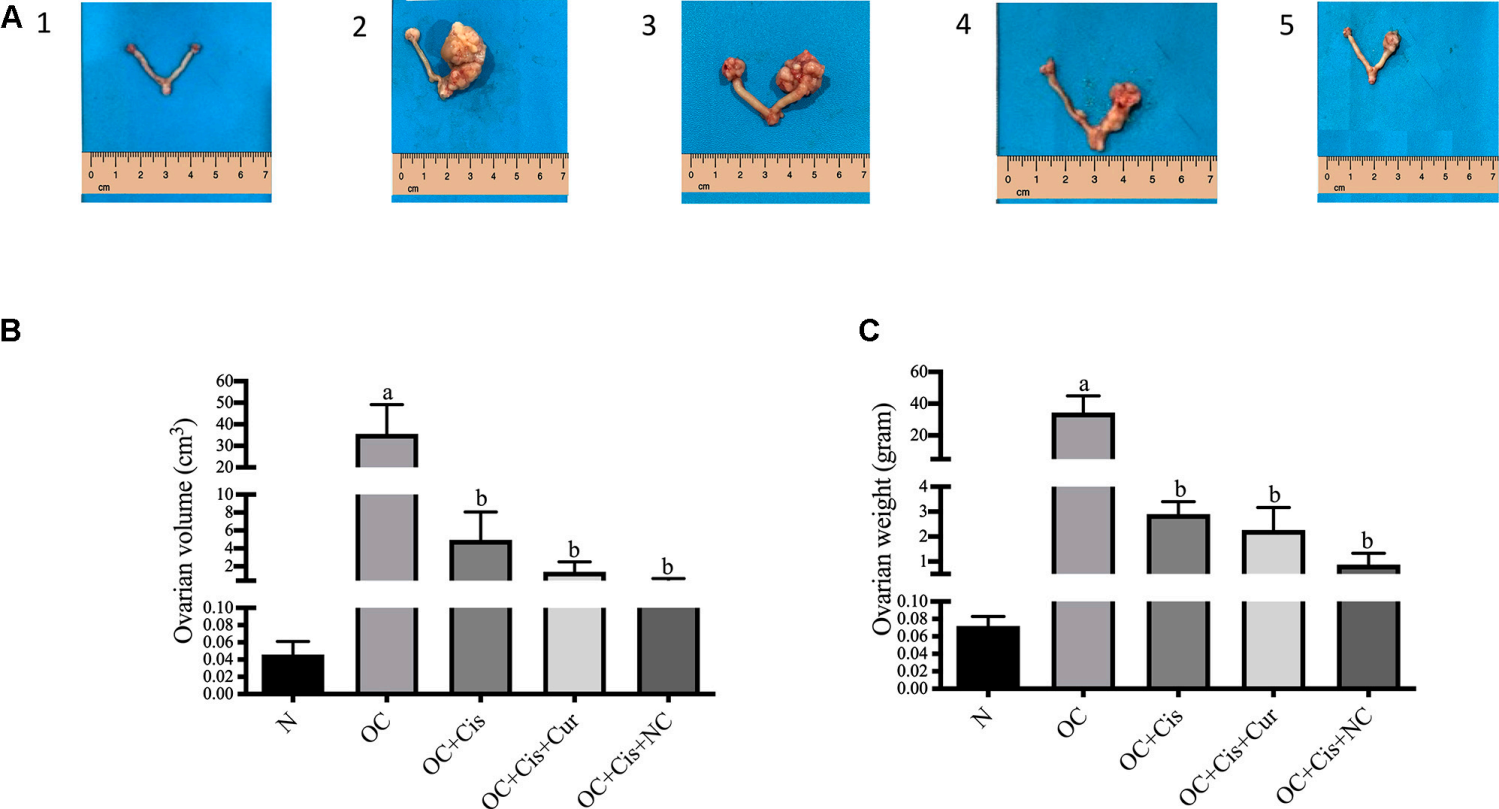
Macroscopic appearance, volume and weight of the rat ovaries after treatment with cisplatin, cisplatin + curcumin or cisplatin + nanocurcumin **(A (1–5))** macroscopic appearance of the rat ovaries **(A-1)** normal/sham **(A-2)** ovarian cancer **(A-3)** ovarian cancer rats treated with cisplatin **(A-4)** ovarian cancer rats treated with cisplatin + curcumin **(A-5)** ovarian cancer rats treated with cisplatin and nanocurcumin **(B)** ovarian volume (cm^3^) and **(C)** ovarian weight **(G)**. Data are presented in mean ± SD and obtained from five rats per group. **(A)** *p* value < 0.05 vs. N (sham); **(B)** *p* value <0.05 vs. N: Normal/Sham; OC: Ovarian *Cancer*; OC + Cis: Ovarian *Cancer* + Cisplatin; OC + Cis + Cur: Ovarian *Cancer* + Cisplatin + Curcumin; OC + Cis + NC: Ovarian *Cancer* + Cisplatin + Nanocurcumin.

We observed a significant reduction in ovarian volume and weight in groups treated with cisplatin, cisplatin + curcumin, and cisplatin + nanocurcumin (Figure 1). However, no notable changes were detected between the three treatment groups.

**FIGURE 2:**
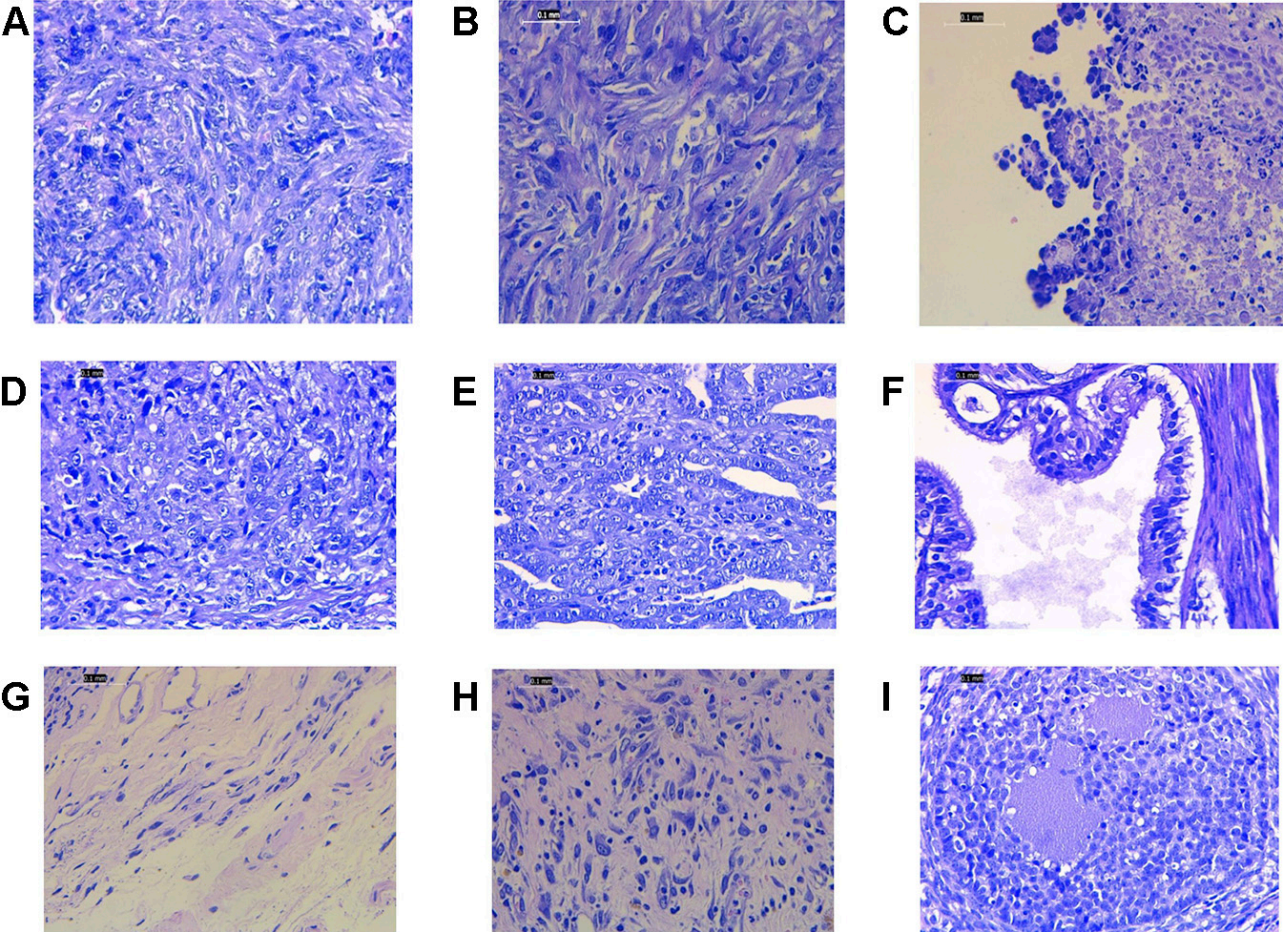
Representative histopathology profiles of the rat ovaries **(A)** poor differentiation adenocarcinoma **(B)** sarcoma **(C)** sarcoma accompanied with budding **(D)** undifferentiated carcinoma **(E)** endometroid adenocarcinoma **(F)** serous mucinosum cystadenoma **(G)** squamous cell carcinoma **(H)** atypical hyperplasia, inflammatory cells without tumors, large nucleoli, irregular shape, stratified, rough chromatin. H&E staining. Magnification at ×400.

Histopathology examinations of the untreated ovarian cancer rats showed low differentiated adenocarcinoma, sarcoma, and sarcoma accompanied by budding. While in the rats treated with cisplatin alone, the majority of histological features showed undifferentiated carcinoma. The group that received cisplatin and curcumin showed histological profiles consisting of endometroid adenocarcinoma, serous, mucinous cystadenoma, and squamous cell carcinoma. In the rats receiving cisplatin and nanocurcumin, ovarian histology showed atypical hyperplasia, inflammatory cells without tumors, large nucleoli, irregular shape, stratified, rough chromatin, and bleeding (Figure 2).

### Changes in the Ki67 and Apoptotic Markers Expressions by Cisplatin + Curcumin/Nanocurcumin

**FIGURE 3:**
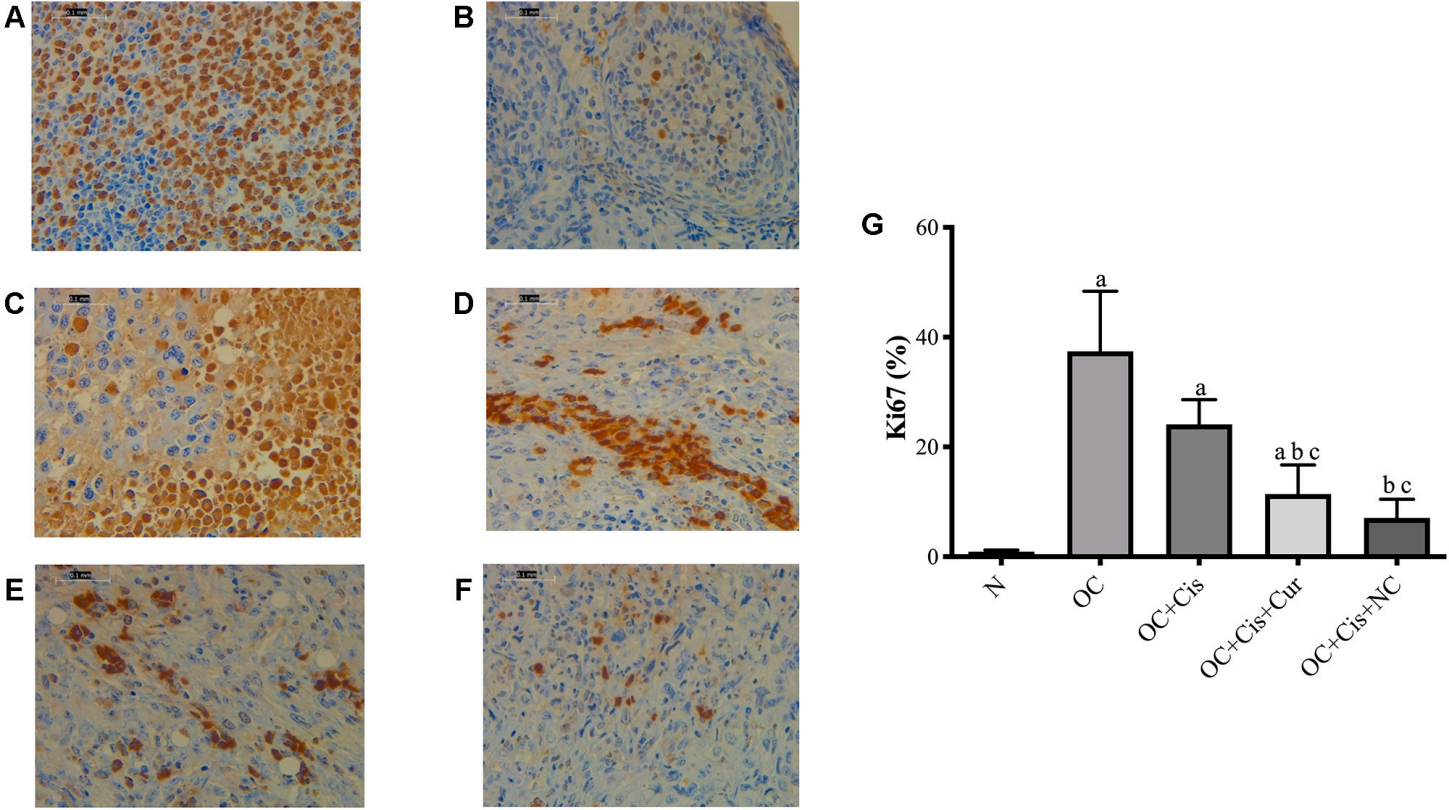
Representative images on the Ki67 protein expressions in the rat ovaries and the result of quantification **(A)** Positive control (reactive tonsil) **(B)** Normal/sham group **(C)** ovarian cancer **(D)** ovarian cancer rats treated with cisplatin **(E)** ovarian cancer rats treated with cisplatin and curcumin; **(F)** ovarian cancer rats treated with cisplatin and nanocurcumin **(G)** The result of Ki67 protein expressions quantifications (calculated using ImageJ) **(A)**–**(F)** Magnification at ×400. **(A)** *p* value < 0.05 vs. N (sham); **(B)** *p* value < 0.05 vs. OC; **(C)** *p* value < 0.05 vs. OC + Cis.

**FIGURE 4:**
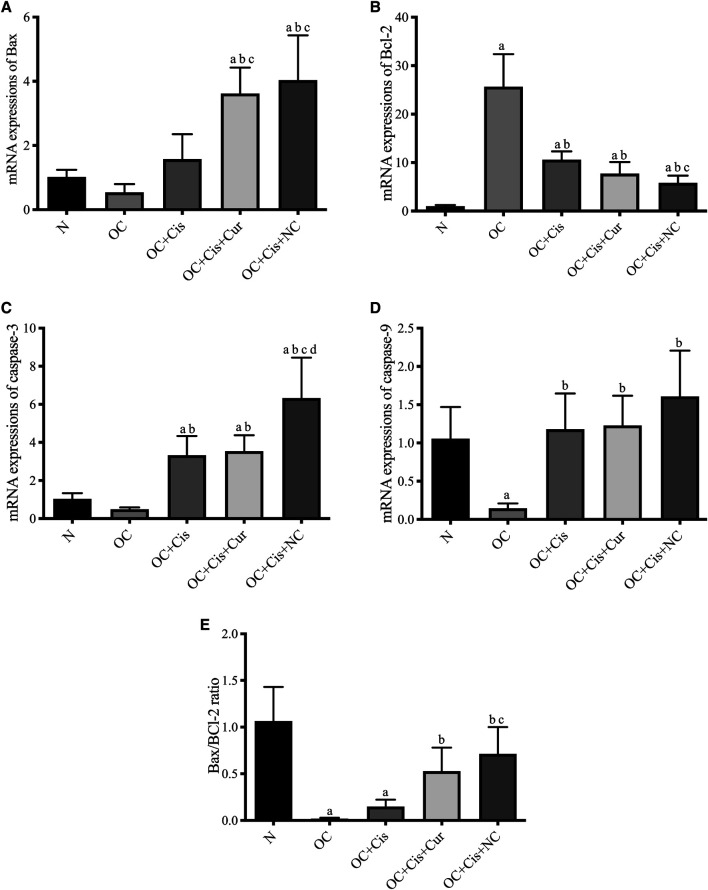
Expressions of apoptosis markers in the rat ovaries after treatment with cisplatin, cisplatin + curcumin or cisplatin + nanocurcumin. **(A)** Bax mRNA expressions; **(B)** BCl2 mRNA expressions; **(C)** Caspase-3 mRNA expressions; **(D)** Caspase-9 mRNA expressions; **(E)** Bax/Bcl-2 ratio. **(A)** *p* value < 0.05 vs. N (sham); **(B)** *p* value < 0.05 vs. OC; **(C)** *p* value < 0.05 vs. OC + Cis; **(D)** *p* value < 0.05 vs. OC + Cis + Cur.

There were decreased expression markers, Ki67, in ovarian rats in the rats treated with cisplatin + curcumin and cisplatin + nanocurcumin (Figure 3). However, no significant difference was shown between the cisplatin-curcumin vs. cisplatin-nanocurcumin group. Furthermore, rats treated with cisplatin-curcumin and cisplatin-nanocurcumin showed a remarkable increase in apoptotic markers, the ratio of Bax/BCl2 mRNA expressions of caspase-3, and caspase-9 (Figure 4).

### Nanocurcumin Causes a Significant Decrease in TGF-β Concentrations and PI3K/Akt Expressions in Ovarian Tissue

The PI3K signal transduction pathway is one of the pathways that play a role in cancer cell growth and resistance to chemotherapy, which is the TGF-β non-Smad pathway. This pathway is one of the signal transduction pathways that play a role in cell proliferation and differentiation. In cancer, this pathway undergoes hyperactivation resulting in excessive Protein kinase B (PKB) (Brown and Toker, 2015). Excessive activity of PKB causes inhibition of pro-apoptotic protein activation, activation of anti-apoptotic proteins, and increased metastasis (Mcdonald, 2008). Inhibition of the TGF-β1/PI3K/Akt pathway represents a potential therapeutic target (Alsina-Sanchis et al., 2016).

**FIGURE 5:**
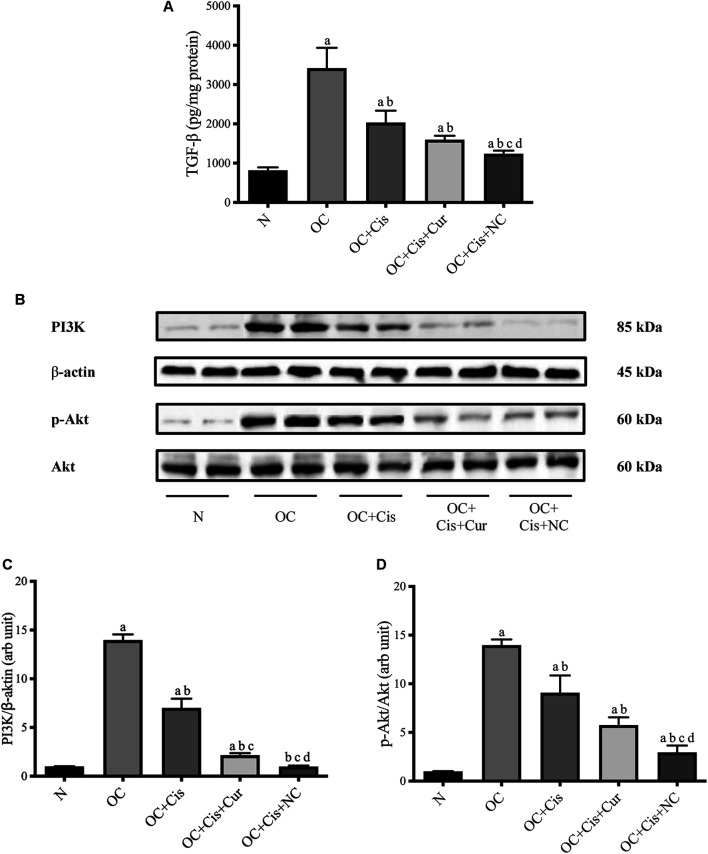
Decreased TGF-β/PI3K/Akt in rat ovaries after treatment with cisplatin or cisplatin + curcumin or cisplatin + nanocurcumin **(A)** Decreased levels of active TGF-β (pg/mg protein) **(B)** Western blot analysis for PI3K (normalized to β-actin as control), and phospho (*p*)-Akt (normalized to Akt as control) **(C)** Downregulation of PI3K/β-actin expressions **(D)** Decreased *p*-Akt/Akt (arb unit). Full blot data are available in Supplementary Material. **(A)** *p* value < 0.05 vs. N (sham) group; **(B)** *p* value < 0.05 vs. OC; **(C)** *p* value < 0.05 vs. OC + Cis; **(D)** *p* value < 0.05 vs. OC + Cis + Cur.

**FIGURE 6:**
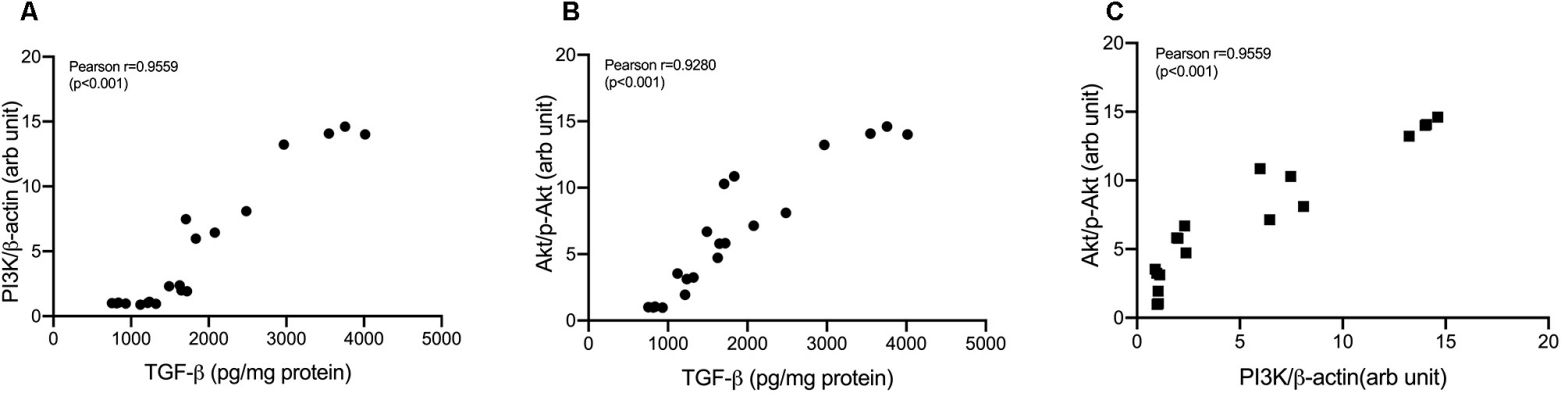
Correlation analysis in TGF-β/PI3K/Akt pathway **(A)** Correlation of TGF-β and PI3K/β-actin **(B)** Correlation of TGF-β and *p*-Akt/Akt **(C)** Correlation of PI3K/β-actin and *p*-Akt/Akt.

Treatment with cisplatin, cisplatin–curcumin, and cisplatin–curcumin resulted in a decreased concentration of ovarian the TGF-β and PI3K expressions and phosphorylation of Akt (Figure 5). The correlations between variables in the TGF-β pathway were shown in Figure 6. TGF-β concentrations very strongly correlate with the PI3K and Akt’s phosphorylation (Pearson correlation: 0.95 and 0.92, respectively). PI3K expressions were also very strongly correlated with Akt’s phosphorylation (Pearson correlation: 0.95). The degree of inhibition was the largest with cisplatin-nanocurcumin, followed by cisplatin-curcumin and cisplatin alone, respectively.

Nanocurcumin causes a substantial reduction in IL-6 concentrations and JAK/STAT3 expressions in ovarian tissues.

Activation of cytokine receptor signals through the JAK-STAT pathway also causes cellular resistance to chemotherapy by accelerating cell proliferation, increasing the regulation of survival factors, inhibiting apoptosis, and activating anti-apoptotic proteins. Inhibition of Activation of STAT3 can provide growth inhibition and induction of apoptosis in tumor cells (Grivennikov et al., 2009; Browning et al., 2018).

**FIGURE 7:**
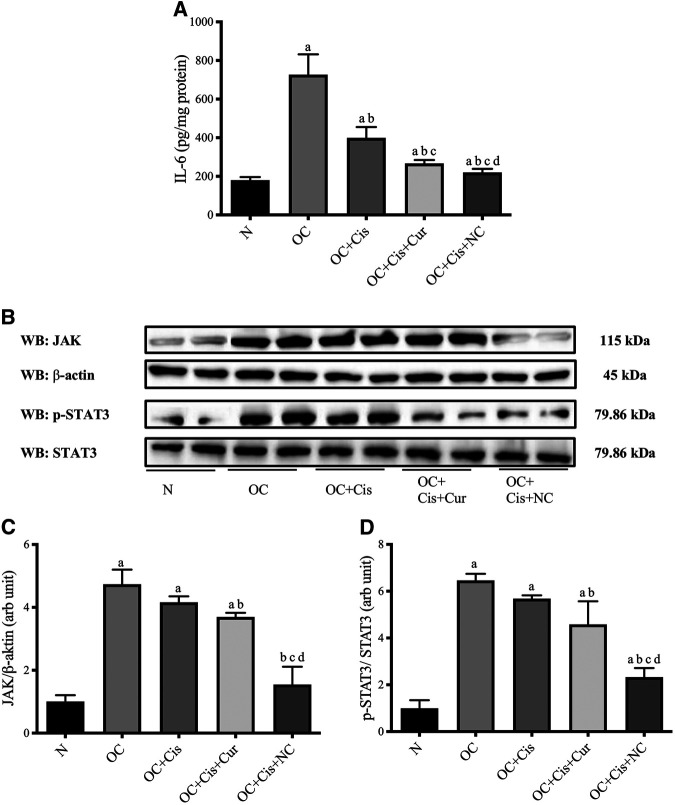
Reduced IL-6 concentrations and JAK/STAT3 expressions in rat ovaries after treatment with cisplatin or cisplatin + curcumin or cisplatin + nanocurcumin **(A)** Decreased levels of IL-6 (pg/mg protein) **(B)** Western blot analysis of JAK (normalized to β-actin as control) and phospho (p)-STAT3 (normalized to STAT3 as control) **(C)** Downregulation of JAK/β-actin protein expressions **(D)** Downregulation of p-STAT3/STAT3 (arb unit).

All treatment groups, cisplatin, cisplatin–curcumin, and cisplatin–nanocurcumin, resulted in decreased concentrations of IL-6 in ovarian tissues, respectively. Consistently, cisplatin-nanocurcumin also showed the highest inhibitory effect on JAK expression and the phosphorylation of STAT3 (Figure 7).

**FIGURE 8:**
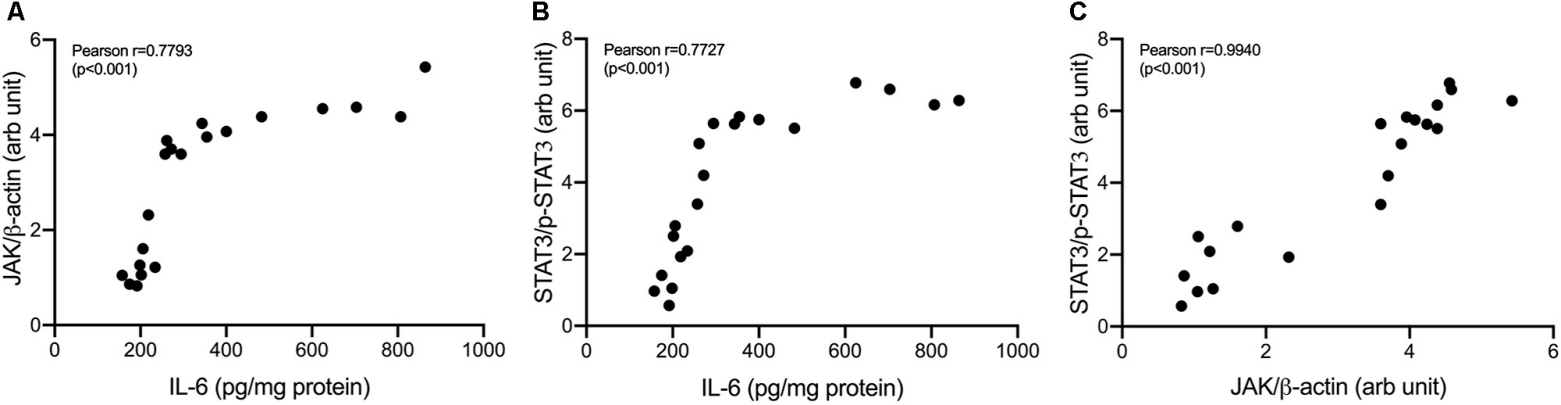
Correlation analysis in IL-6/JAK/STAT3 pathway. **(A)** Correlation of IL-6 and JAK/β-actin **(B)** Correlation of IL-6 and p-STAT3/STAT3 **(C)** Correlation of JAK/β-actin and p-STAT3/STAT3.

Correlations between variables in IL-6/JAK/STAT3 pathways were shown in Figure 8. IL-6 concentrations strongly correlate with JAK expressions and STAT3 phosphorylation (Pearson correlation: 0.78 and 0.77, respectively). JAK expressions were shown to be strongly correlated with STAT3 phosphorylation (Pearson correlation: 0.94).

## Discussion

Studies have shown that the efficacy of chemotherapy-induced tumor growth suppression and apoptosis in ovarian cancer cell lines can be improve by anticancer agent combinations (Markman, 2019). Curcumin has demonstrated a multifunctional mechanism of anticancer activity in primary epithelial ovarian cancer resistant to platinum and in multidrug-resistant cancer cells (Terlikowska et al., 2014). Its low bioavailability however limits the efficacy of curcumin. Curcumin formulation in the form of nanoparticles could improve its bioavailability and efficacy (Shanmugam et al., 2015; Karthikeyan et al., 2020).

Our study demonstrated that curcumin and nanocurcumin can potentiate cisplatin’s anticancer effect by reducing the volume and weight of the ovarian tumor. Our result indicates no notable differences in the efficacy of curcumin vs. nanocurcumin on the ovarian morphological outcomes. However, nanocurcumin resulted in better outcomes when compared with curcumin in Ki-67 expressions, known prognostic markers, and predictors for chemotherapy outcomes in ovarian cancer (Mahadevappa et al., 2017; Grabowski et al., 2020). We believe that a longer duration of treatment with nanocurcumin might result in better overall outcomes and survival in our ovarian cancer model.

Ki-67 is a nuclear protein used as a proliferation marker (Mahadevappa et al., 2017; Qiu et al., 2019). However, in our result, we also observed cytoplasmic staining of Ki-67. The antibody used to determine the Ki-67 expressions in the cells was monoclonal and used according to manufacturers’ protocols. However, human tonsil tissue used as positive control also showed cytoplasmic staining and resembled the manufacturer’s sample photo. Studies had sometimes discovered unusual Ki-67 immunoreactivity in the cytoplasm membranous (Faratian et al., 2009; Kim et al., 2013). ln human breast cancer cells, it was reported that the cytoplasmic staining of Ki-67 may be an essential yet separate prognostic marker in some subtypes of breast cancer.

Furthermore, our findings were confirmed by an enhancement of apoptosis evidenced from increased ratio of Bax/Bcl-2, caspase-9, and caspase-3 mRNA expressions in cisplatin and curcumin or nanocurcumin combinations. Cisplatin-nanocurcumin combination significantly increased apoptotic markers when compared with cisplatin-curcumin, following the decreased proliferation marker, Ki-67. Our results indicate that the addition of nanocurcumin to cisplatin can be used as an efficient strategy to induce cancer cell death. With molecular-targeted therapy, treatments for ovarian cancer are currently being developed to improve conventional chemotherapy (Melichar et al., 2006). At the molecular level, curcumin targets numerous pathways, highlighting its ability to inhibit multistep carcinogenesis (Shanmugam et al., 2015).

We also observed a remarkable impact of nanocurcumin in inhibiting TGF-β and IL-6 pathways. Both pathways are responsible for developing malignant growth in ovarian cancer (Alsina-Sanchis et al., 2016; Browning et al., 2018).

A molecularly targeted combination of anticancer agents provides a new approach to improving cancer effectiveness. To optimize cisplatin’s efficacy, the addition of agents that work on different pathway target can increase the potential for chemotherapy and increase the survival rate in advanced ovarian cancer (Yunos et al., 2011). In this study, we demonstrated that cisplatin plus nanocurcumin has a synergistic effect by generating antiproliferation and apoptosis in ovarian cancer animal models by downregulation of PI3K/AKT and JAK/STAT3 signaling pathway.

PI3K/Akt is downstream of TGF-β non-Smad pathway. PI3K/Akt is a well-known major regulatory signaling pathways that modulate the progression of tumor cell development through apoptotic inhibitory activity, increased cell proliferation, and stimulation of angiogenesis, invasion, and metastasis (Bondar et al., 2002). In our study, the ovarian cancer animal model has resulted in an increased expression of TGF-β. TGF-β is a vital factor in follicle development (Alsina-Sanchis et al., 2016). Our result showed that the combination of cisplatin and nanocurcumin strongly reduced TGF-β concentrations, better than cisplatin only or cisplatin-curcumin. Our work was in line with a study by Alsina et al., showing that inhibition of TGF-β can block tumor growth in orthotopic preclinical models of ovarian cancer (PDX) (Alsina-Sanchis et al., 2016). A recent study reported that a combination of cisplatin and TGF-β inhibitor produced a better antiproliferative effect than single chemotherapy in an ovarian cancer xenograft model. Blocking TGF-β signaling is a therapeutic approach to ovarian cancer that will provide opportunities for these patients by involving the role that TGF-β plays in the proliferation of ovarian cancer. A better prognosis was associated with a low level of TGF-β expression in advanced ovarian tumors (Komiyama et al., 2011).

The TGF-β pathway triggers well-known intracellular pathways, including phosphatidylinositol-3-kinase/Akt (Elfiky et al., 2011). Our correlation analysis confirmed the very strong correlation between TGF-β and PI3K and phosphorylation of Akt. Cisplatin and nanocurcumin combination efficiently inhibit TGF-β, PI3K, and Akt phosphorylation. These findings resulted from the PI3K/Akt inhibition of cisplatin and nanocurcumin or curcumin. Our finding supports curcumin and nanocurcumin as PI3K/Akt inhibitors, along with other agents such as LY294002 and Wortmannin (Ng et al., 2001; Bondar et al., 2002).

In addition to TGF-β, interleukin-6 (IL-6) also plays an essential role in ovarian carcinogenesis. IL-6 works by binding with cytokine receptors, which can trigger signaling through the Jak/STAT/Ras/MAPK and PI3K/Akt pathway (Browning et al., 2018; Taher et al., 2018). IL-6 is one of the crucial regulators that help communicate between tumor cells and their microenvironment. Increased IL-6 production in tumor growth and development has been demonstrated in many tumors (Grivennikov et al., 2009). The elevated IL-6 levels activate STAT3 in surrounding tumors. Active tumor cells will produce even more IL-6, promoting the tumor environment with inflammatory mediators that support tumor growth and metastasis (Yu et al., 2009). Here, we demonstrate that cisplatin and nanocurcumin treatment suppressed the JAK/STAT3 pathway and reduced IL-6 expression, suggesting that the JAK/STAT3 pathway mediates the autocrine production of IL-6 in the ovarian cancer model. By suppressing STAT3 phosphorylation, STAT3 activation is reduced, IL-6 production decreases, thus inhibiting tumor growth and ovarian metastases. Our findings suggest a strong correlation between IL-6 and JAK expressions and STAT3 phosphorylation.

STAT3 is activated constitutively in many types of tumors, including ovarian cancer, and this activation promotes accelerated cell proliferation, increased regulation of survival factors, and activation of anti-apoptotic proteins (Yu and Jove, 2004). Our results showed that the inhibition of JAK and STAT3 phosphorylation was mainly mediated by curcumin and nanocurcumin. Our study showed that cisplatin had a limited effect on JAK and STAT3 (Figure 6). The phosphorylation of STAT3 investigated in our study was pSTAT3^Tyr705^. Phosphorylation of STAT3 in the tyrosine 705 domain has been found to promote epithelial-mesenchymal transition and metastasis in cancer cells. The phosphorylation of STAT3^Tyr705^ was also found to activate further MAPK and PI3K/Akt, which lead to cisplatin resistance (Liang et al., 2019; Wu et al., 2019). Therefore, curcumin or nanocurcumin to cisplatin will add benefit by providing an alternative inhibitory pathway to cisplatin in carcinogenesis, thereby preventing cisplatin resistance.

The present study establishes curcumin and nanocurcumin as adjunctive treatment to cisplatin, which resulted in potent inhibition of proliferation marker, induction of apoptosis, and inhibition of tumor growth in rat ovarian cancer. The lack of inhibitory effect of cisplatin on JAK and STAT3 phosphorylation brought curcumin and nanocurcumin as a potent anticancer candidate especially when JAK/STAT3 is overexpressed. Our findings confirm other studies that showed inhibition of JAK and STAT3 protein activation results in growth inhibition and apoptosis in tumor cells both *in vitro* and *in vivo* (Lin et al., 2009; Singh et al., 2009).

Together, the present study has established the role of nanocurcumin as adjunctive treatment to cisplatin in the ovarian cancer model through inhibition of TGF-β/PI3K/Akt and IL-6/JAK/STAT3 pathways. To further support our findings, another study to confirm the preclinical activity of the drug using a mice tumor xenograft might be needed, as suggested by several studies (Hernandez et al., 2016; Maru and Hippo, 2019; Huang et al., 2020a).

Curcumin has been shown as a very safe compound in high doses. Clinical trials in various cancers have used up to 3,000 mg per day. Despite using a high amount, clinical trials with curcumin still showed unsatisfactory efficacy results due to its low bioavailability, poor absorption, rapid elimination, or low target organ concentration. (Cruz-Correa et al., 2018; Choi et al., 2019; Howells et al., 2019). Nanoparticle formulations of curcumin were proven to increase the bioavailability of curcumin. Additionally, Nanocurcumin used in our study has been shown to increase its plasma concentrations, as shown by AUC up to 20-folds (Arozal et al., 2020). Our findings demonstrated that nanocurcumin improves the anticancer activity of cisplatin better than conventional curcumin. To the best of our knowledge, no clinical trials on curcumin have been conducted. The present results of cisplatin and nanocurcumin combination warrant further clinical investigations to evaluate patients’ efficacy and safety.

## Conclusion

Our study demonstrates that nanocurcumin potentiates the anticancer effect of cisplatin by reducing tumor volume and weight. The activity of nanocurcumin is mediated by inhibiting TGF-β and IL-6. Furthermore, nanocurcumin downregulates PI3K/Akt and JAK/STAT3 signaling pathways, which at least in part have a role in preventing further tumor proliferation and growth. Our findings provide a new insight into the molecular mechanism of nanocurcumin and its therapeutic potential to treat ovarian cancer.

## Data Availability Statement

The original contributions presented in the study are included in the article/Supplementary Material, further inquiries can be directed to the corresponding author.

## Ethics Statement

The animal study was reviewed and approved by Health Research Ethics Committee Faculty of Medicine Universitas Indonesia.

## Author Contributions

NS, ML, and WA, designed the experiment, analyzed the data, and write the manuscript. NS, DR, and PW experimented and contributed to the data analysis. All of the authors approved the final manuscript.

## Funding

This research was supported by a research grant from the Penelitian Dasar Unggulan Perguruan Tinggi (PDUPT) 2020 from the Ministry of Research, Technology and Higher Education, Republic of Indonesia.

## Conflict of Interest

The authors declare that the research was conducted in the absence of any commercial or financial relationships that could be construed as a potential conflict of interest.
