# Supplementary material for: Curcumin Nanoparticle Enhances the Anticancer Effect of Cisplatin by Inhibiting PI3K/AKT and JAK/STAT3 Pathway in Rat Ovarian Carcinoma Induced by DMBA
Source: Front Pharmacol. 2021 Jan 18;11:603235. doi: 10.3389/fphar.2020.603235 (PMC7848208; doi:10.3389/fphar.2020.603235)
Supplement: Supplementary file 1 [file datasheet1.pdf]

β-actin

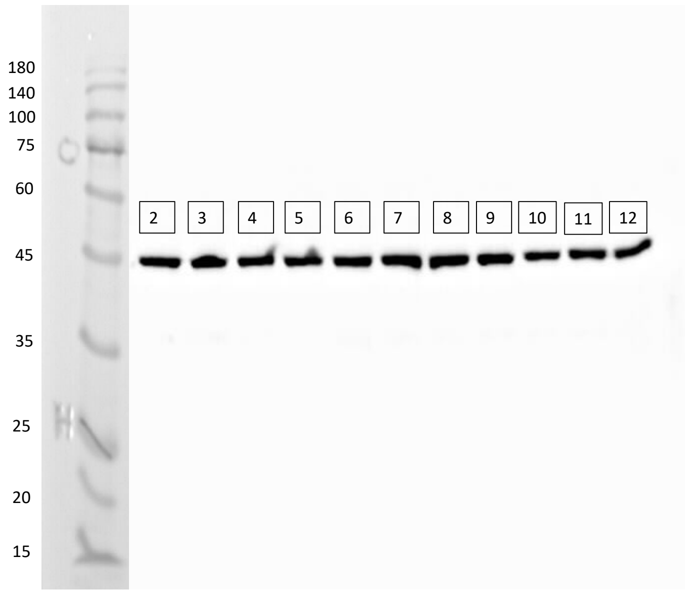

WB: Beta aktin 45 kDa
(Monoclonal, CST)

1. marker
2. Ca Ovarium (A1)
3. Ca Ovarium (A2)
4. Ca Ov. + Cisplatin (B1)
5. Ca Ov. + Cisplatin (B2)
6. Ca Ov. + Cisplatin + Kurkumin (C1)
7. Ca Ov. + Cisplatin + Kurkumin (C2)
8. Ca Ov. + Cisplatin + Nanokurkumin (D1)
9. Ca Ov. + Cisplatin + Nanokurkumin (D2)
10. Sham (E1)
11. Sham (E2)
12. Sham (E5)

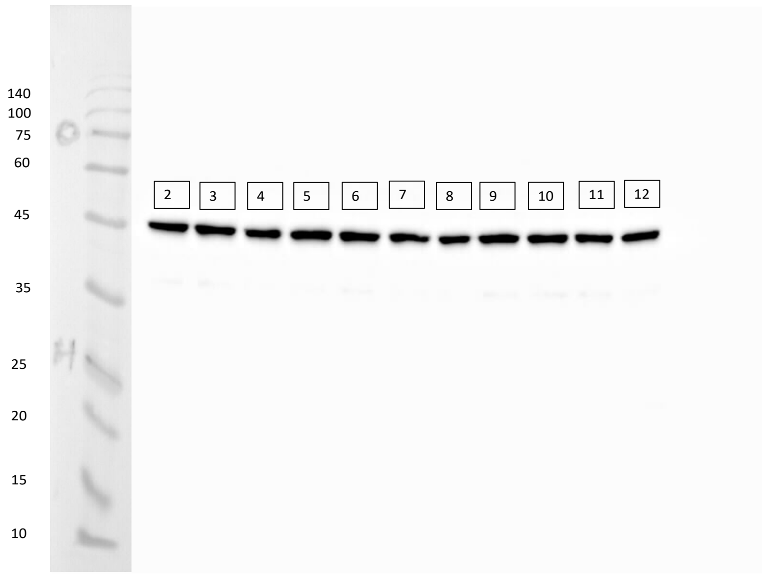

WB: Beta aktin 45 kDa
(Monoclonal, CST)

1. marker
2. Ca Ovarium (A3)
3. Ca Ovarium (A4)
4. Ca Ov. + Cisplatin (B3)
5. Ca Ov. + Cisplatin (B4)
6. Ca Ov. + Cisplatin + Kurkumin (C3)
7. Ca Ov. + Cisplatin + Kurkumin (C4)
8. Ca Ov. + Cisplatin + Nanokurkumin (D3)
9. Ca Ov. + Cisplatin + Nanokurkumin (D4)
10. Sham (E3)
11. Sham (E4)
12. Sham (E6)

PI3K

WB: PI3K 85 kDa
(Monoclonal, CST)

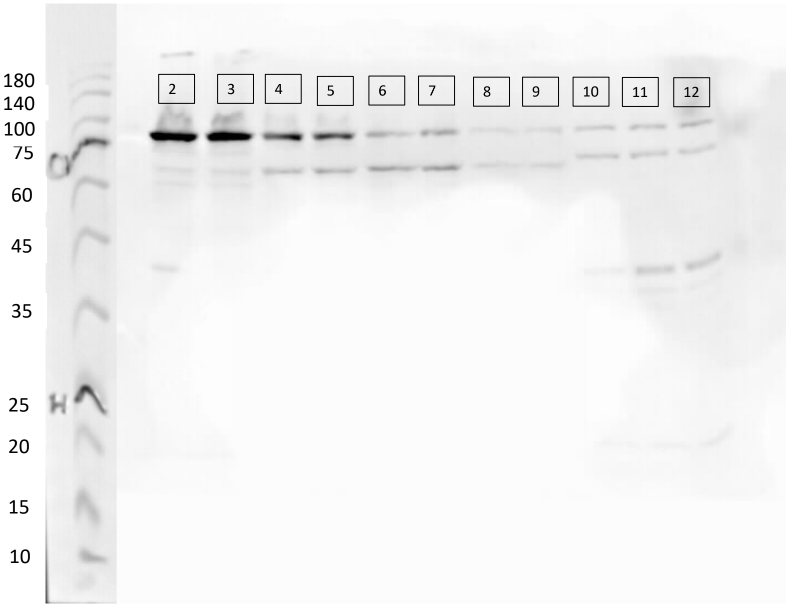

1. marker
2. Ca Ovarium (A1)
3. Ca Ovarium (A2)
4. Ca Ov. + Cisplatin (B1)
5. Ca Ov. + Cisplatin (B2)
6. Ca Ov. + Cisplatin + Kurkumin (C1)
7. Ca Ov. + Cisplatin + Kurkumin (C2)
8. Ca Ov. + Cisplatin + Nanokurkumin (D1)
9. Ca Ov. + Cisplatin + Nanokurkumin (D2)
10. Sham (E1)
11. Sham (E2)
12. Sham (E5)

6

WB: PI3K 85 kDa
(Monoclonal, CST)

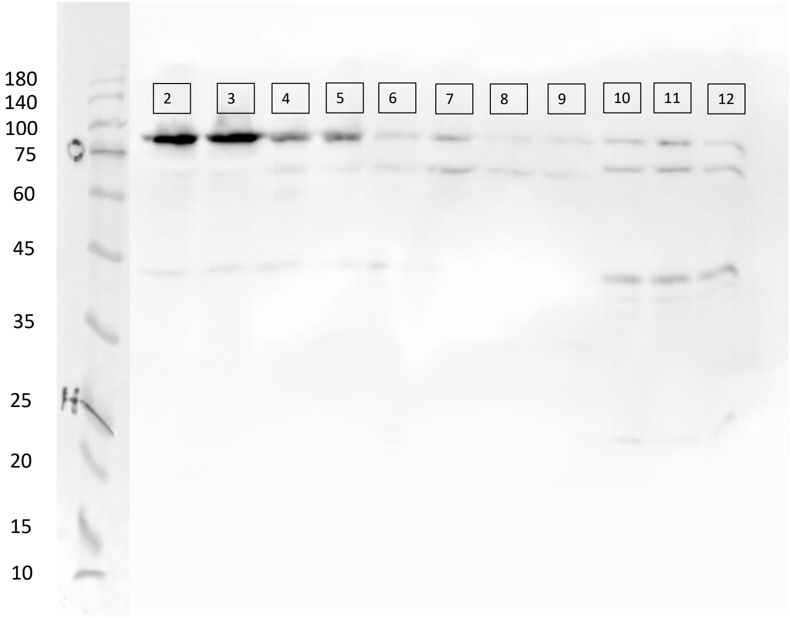

1. marker
2. Ca Ovarium (A3)
3. Ca Ovarium (A4)
4. Ca Ov. + Cisplatin (B3)
5. Ca Ov. + Cisplatin (B4)
6. Ca Ov. + Cisplatin + Kurkumin (C3)
7. Ca Ov. + Cisplatin + Kurkumin (C4)
8. Ca Ov. + Cisplatin + Nanokurkumin (D3)
9. Ca Ov. + Cisplatin + Nanokurkumin (D4)
10. Sham (E3)
11. Sham (E4)
12. Sham (E6)

8

Akt

WB: Akt 60 kDa
(Monoclonal, CST)

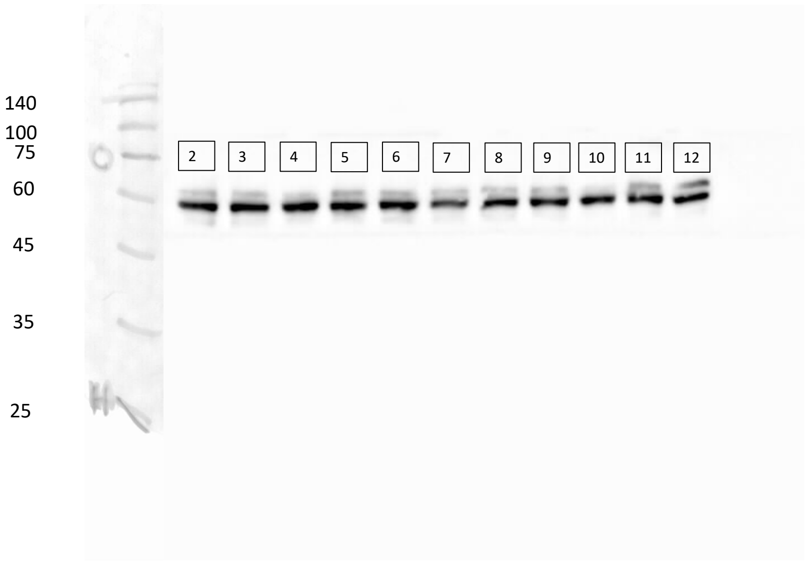

1. marker
2. Ca Ovarium (A1)
3. Ca Ovarium (A2)
4. Ca Ov. + Cisplatin (B1)
5. Ca Ov. + Cisplatin (B2)
6. Ca Ov. + Cisplatin + Kurkumin (C1)
7. Ca Ov. + Cisplatin + Kurkumin (C2)
8. Ca Ov. + Cisplatin + Nanokurkumin (D1)
9. Ca Ov. + Cisplatin + Nanokurkumin (D2)
10. Sham (E1)
11. Sham (E2)
12. Sham (E5)

WB: Akt 60 kDa
(Monoclonal, CST)

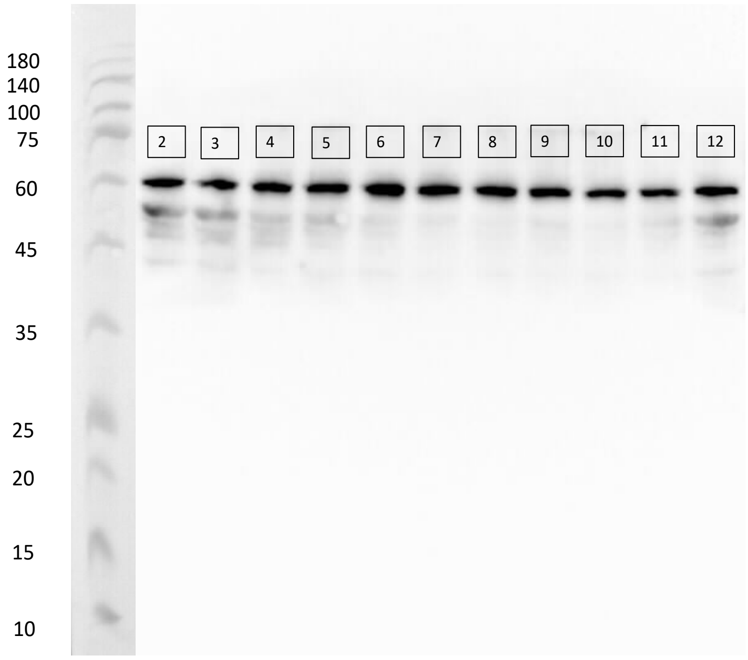

1. marker
2. Ca Ovarium (A3)
3. Ca Ovarium (A4)
4. Ca Ov. + Cisplatin (B3)
5. Ca Ov. + Cisplatin (B4)
6. Ca Ov. + Cisplatin + Kurkumin (C3)
7. Ca Ov. + Cisplatin + Kurkumin (C4)
8. Ca Ov. + Cisplatin + Nanokurkumin (D3)
9. Ca Ov. + Cisplatin + Nanokurkumin (D4)
10. Sham (E3)
11. Sham (E4)
12. Sham (E6)

p-Akt

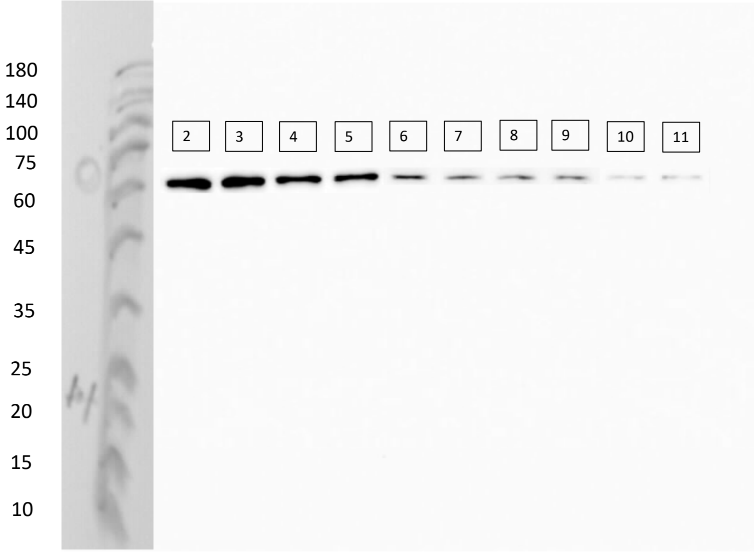

WB: p-Akt 60 kDa
(Monoclonal, CST)

1. marker
2. Ca Ovarium (A1)
3. Ca Ovarium (A2)
4. Ca Ov. + Cisplatin (B1)
5. Ca Ov. + Cisplatin (B2)
6. Ca Ov. + Cisplatin + Kurkumin (C1)
7. Ca Ov. + Cisplatin + Kurkumin (C2)
8. Ca Ov. + Cisplatin + Nanokurkumin (D1)
9. Ca Ov. + Cisplatin + Nanokurkumin (D2)
10. Sham (E1)
11. Sham (E2)

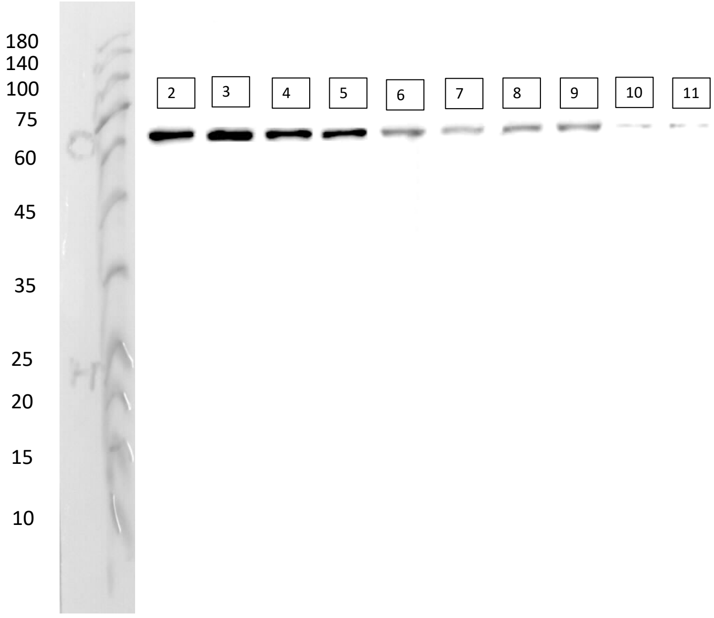

WB: p-Akt 60 kDa
(Monoclonal, CST)

1. marker
2. Ca Ovarium (A3)
3. Ca Ovarium (A4)
4. Ca Ov. + Cisplatin (B3)
5. Ca Ov. + Cisplatin (B4)
6. Ca Ov. + Cisplatin + Kurkumin (C3)
7. Ca Ov. + Cisplatin + Kurkumin (C4)
8. Ca Ov. + Cisplatin + Nanokurkumin (D3)
9. Ca Ov. + Cisplatin + Nanokurkumin (D4)
10. Sham (E3)
11. Sham (E4)

JAK

WB: JAK 115 kDa
(Monoclonal, CST)

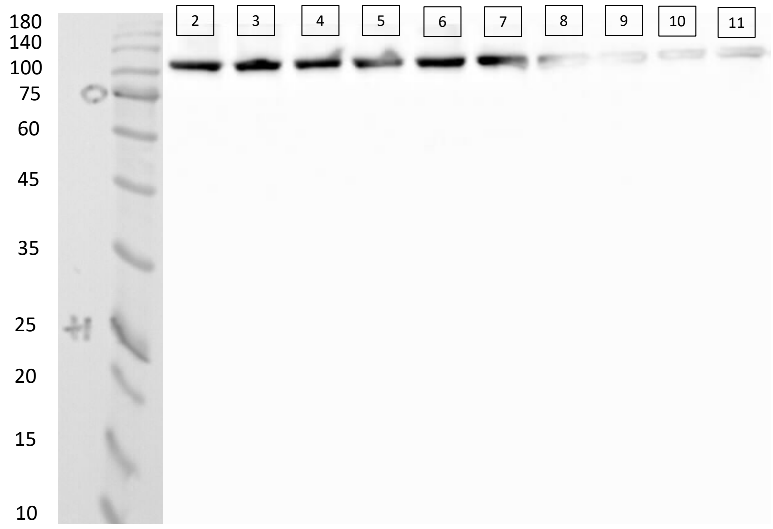

1. marker
2. Ca Ovarium (A1)
3. Ca Ovarium (A2)
4. Ca Ov. + Cisplatin (B1)
5. Ca Ov. + Cisplatin (B2)
6. Ca Ov. + Cisplatin + Kurkumin (C1)
7. Ca Ov. + Cisplatin + Kurkumin (C2)
8. Ca Ov. + Cisplatin + Nanokurkumin (D1)
9. Ca Ov. + Cisplatin + Nanokurkumin (D2)
10. Sham (E1)
11. Sham (E2)

WB: JAK 115 kDa
(Monoclonal, CST)

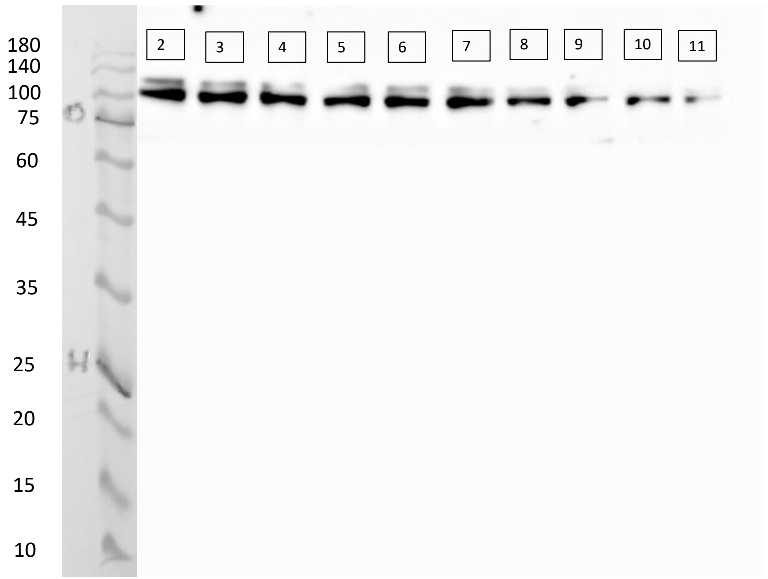

1. marker
2. Ca Ovarium (A3)
3. Ca Ovarium (A4)
4. Ca Ov. + Cisplatin (B3)
5. Ca Ov. + Cisplatin (B4)
6. Ca Ov. + Cisplatin + Kurkumin (C3)
7. Ca Ov. + Cisplatin + Kurkumin (C4)
8. Ca Ov. + Cisplatin + Nanokurkumin (D3)
9. Ca Ov. + Cisplatin + Nanokurkumin (D4)
10. Sham (E3)
11. Sham (E4)

STAT3

WB: STAT 79,86 kDa
(Monoclonal, CST)

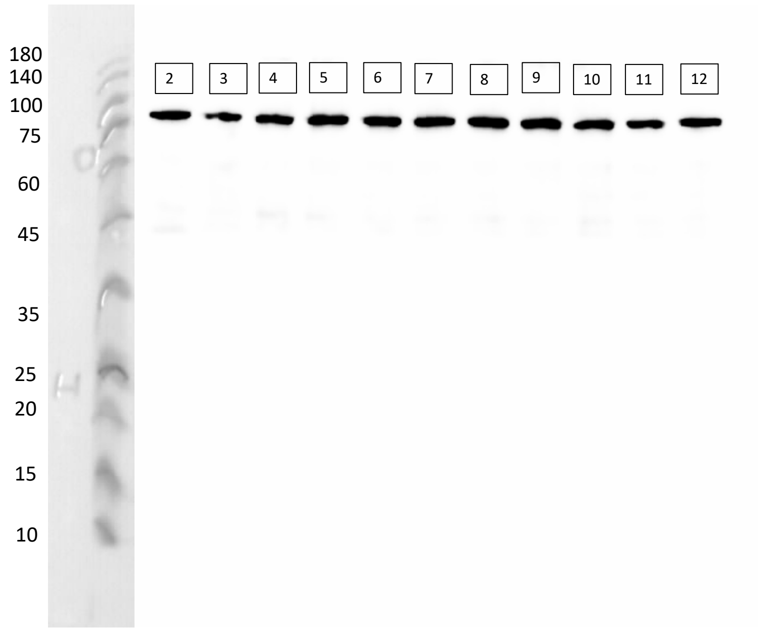

1. marker
2. Ca Ovarium (A1)
3. Ca Ovarium (A2)
4. Ca Ov. + Cisplatin (B1)
5. Ca Ov. + Cisplatin (B2)
6. Ca Ov. + Cisplatin + Kurkumin (C1)
7. Ca Ov. + Cisplatin + Kurkumin (C2)
8. Ca Ov. + Cisplatin + Nanokurkumin (D1)
9. Ca Ov. + Cisplatin + Nanokurkumin (D2)
10. Sham (E1)
11. Sham (E2)
12. Sham (E5)

WB: STAT 79,86 kDa
(Monoclonal, CST)

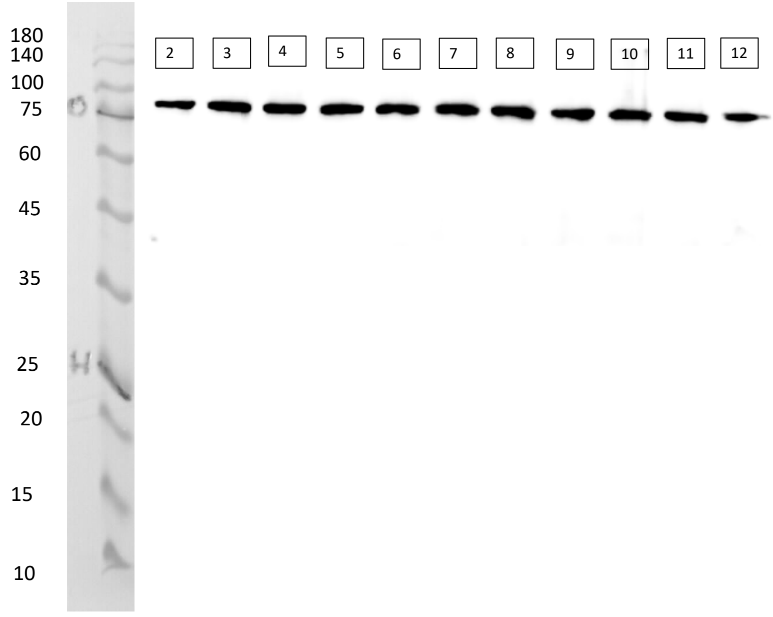

1. marker
2. Ca Ovarium (A3)
3. Ca Ovarium (A4)
4. Ca Ov. + Cisplatin (B3)
5. Ca Ov. + Cisplatin (B4)
6. Ca Ov. + Cisplatin + Kurkumin (C3)
7. Ca Ov. + Cisplatin + Kurkumin (C4)
8. Ca Ov. + Cisplatin + Nanokurkumin (D3)
9. Ca Ov. + Cisplatin + Nanokurkumin (D4)
10. Sham (E3)
11. Sham (E4)
12. Sham (E6)

p-STAT

WB: p-STAT 79,86 kDa
(Monoclonal, CST)

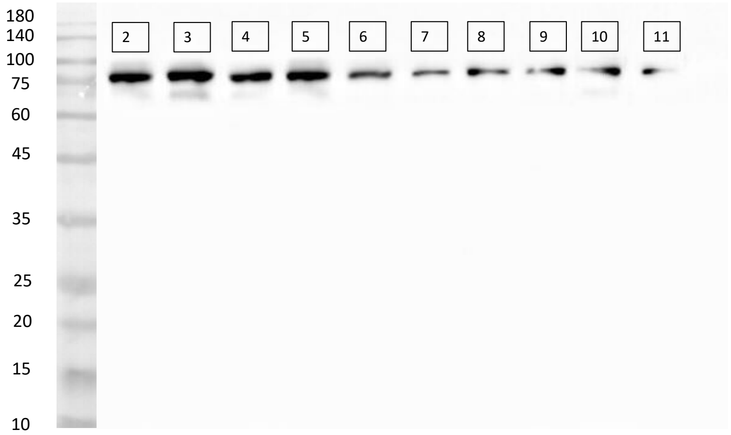

1. marker
2. Ca Ovarium (A3)
3. Ca Ovarium (A4)
4. Ca Ov. + Cisplatin (B3)
5. Ca Ov. + Cisplatin (B4)
6. Ca Ov. + Cisplatin + Kurkumin (C3)
7. Ca Ov. + Cisplatin + Kurkumin (C4)
8. Ca Ov. + Cisplatin + Nanokurkumin (D3)
9. Ca Ov. + Cisplatin + Nanokurkumin (D4)
10. Sham (E3)
11. Sham (E4)

WB: p-STAT 79,86 kDa
(Monoclonal, CST)

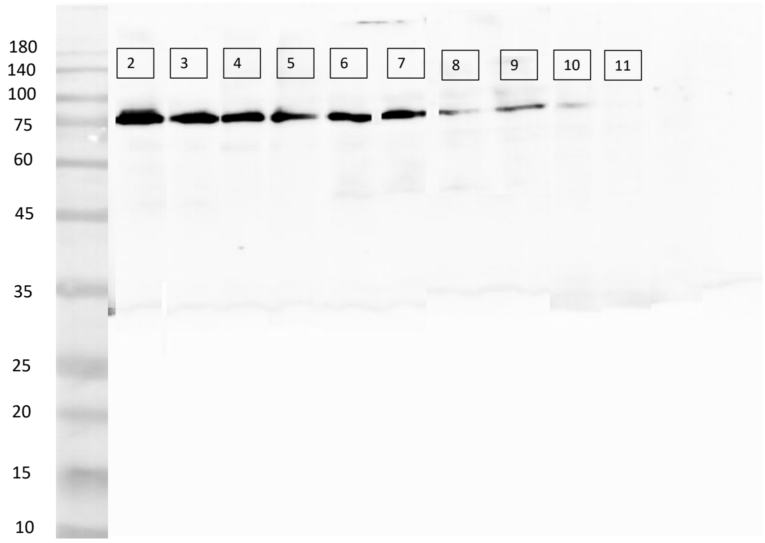

1. marker
2. Ca Ovarium (A3)
3. Ca Ovarium (A4)
4. Ca Ov. + Cisplatin (B3)
5. Ca Ov. + Cisplatin (B4)
6. Ca Ov. + Cisplatin + Kurkumin (C3)
7. Ca Ov. + Cisplatin + Kurkumin (C4)
8. Ca Ov. + Cisplatin + Nanokurkumin (D3)
9. Ca Ov. + Cisplatin + Nanokurkumin (D4)
10. Sham (E3)
11. Sham (E4)
