# Supplementary figures and images for: Curcumin Nanoparticle Enhances the Anticancer Effect of Cisplatin by Inhibiting PI3K/AKT and JAK/STAT3 Pathway in Rat Ovarian Carcinoma Induced by DMBA
Source: Front Pharmacol. 2021 Jan 18;11:603235. doi: 10.3389/fphar.2020.603235 (PMC7848208; doi:10.3389/fphar.2020.603235)

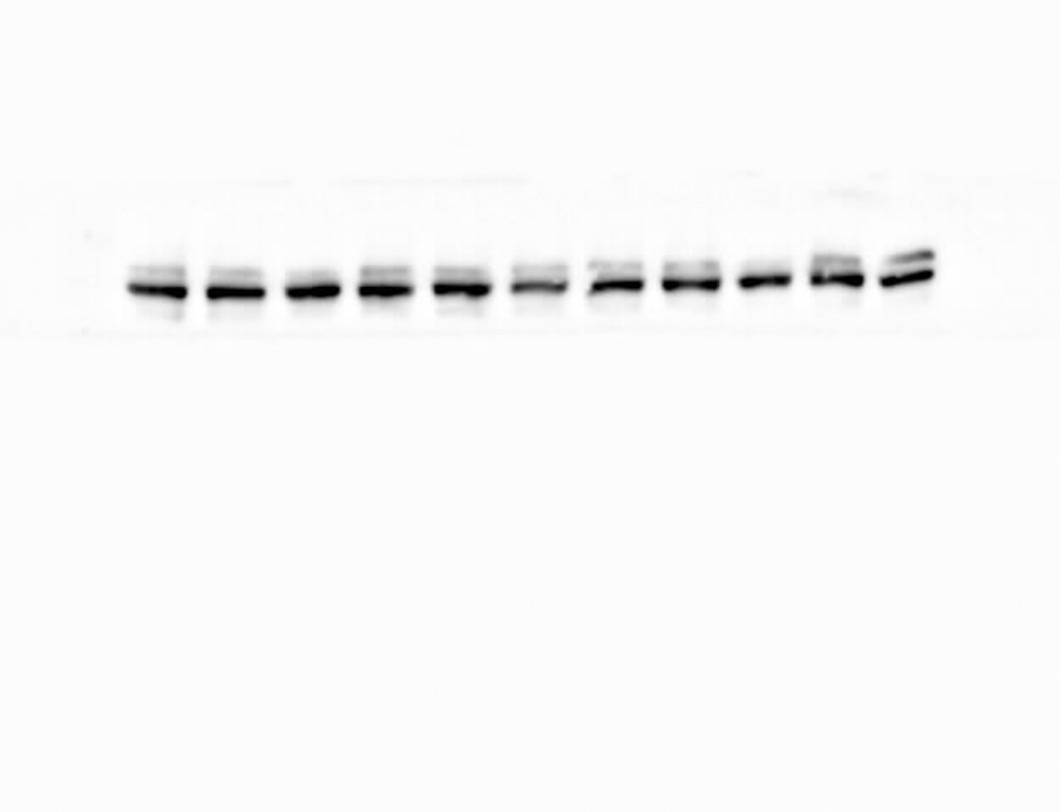

Supplement: Supplementary file 2 [file datasheet2.zip › Akt.jpg]

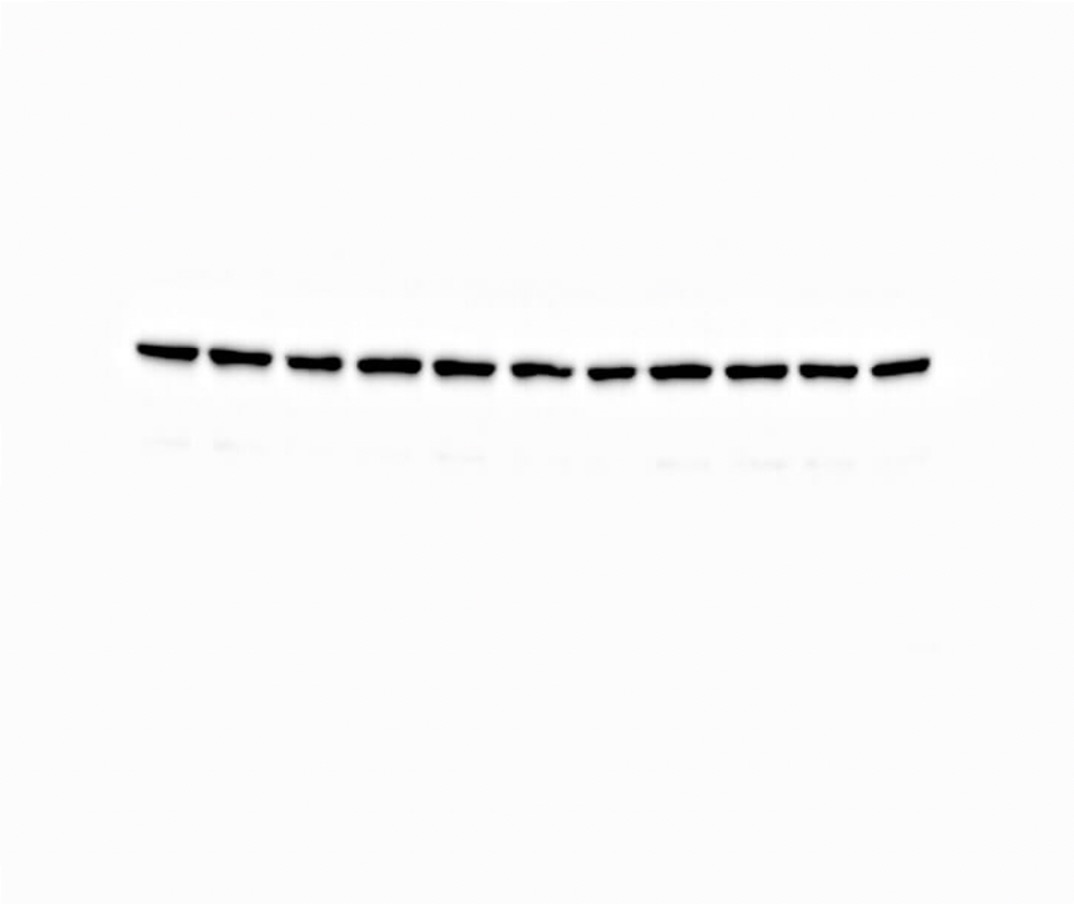

Supplement: Supplementary file 2 [file datasheet2.zip › Beta Actin 2.jpg]

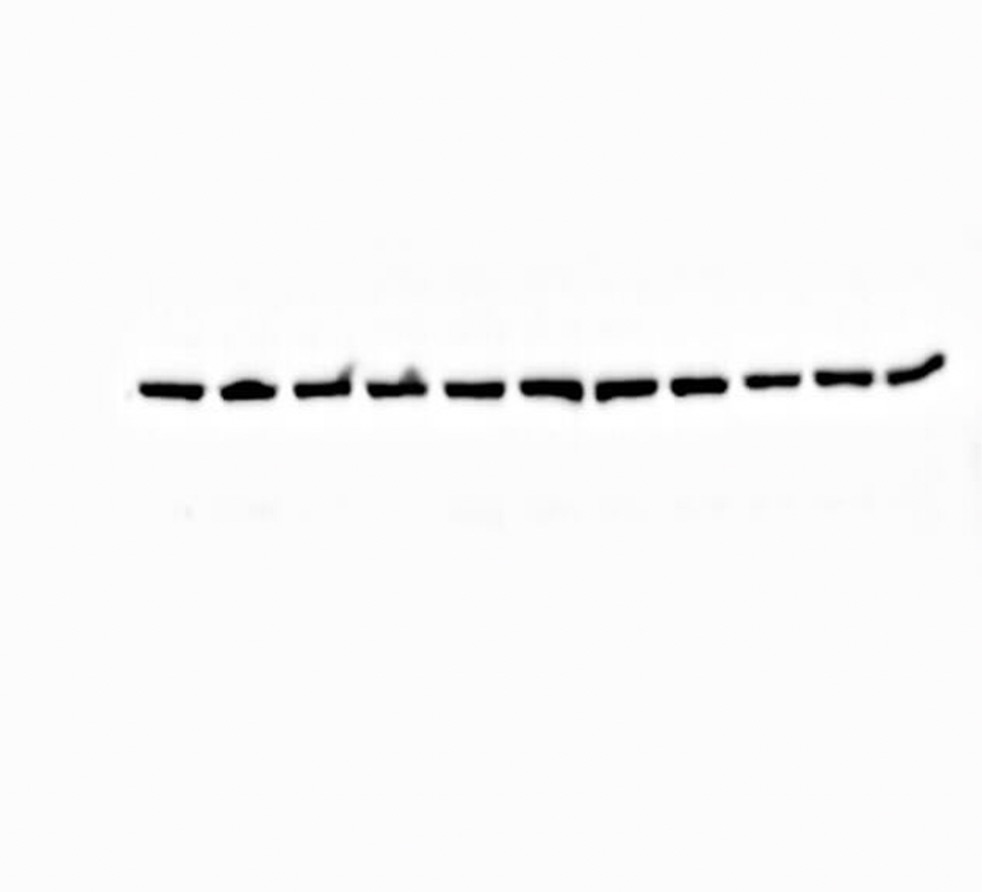

Supplement: Supplementary file 2 [file datasheet2.zip › Beta Aktin 1.jpg]

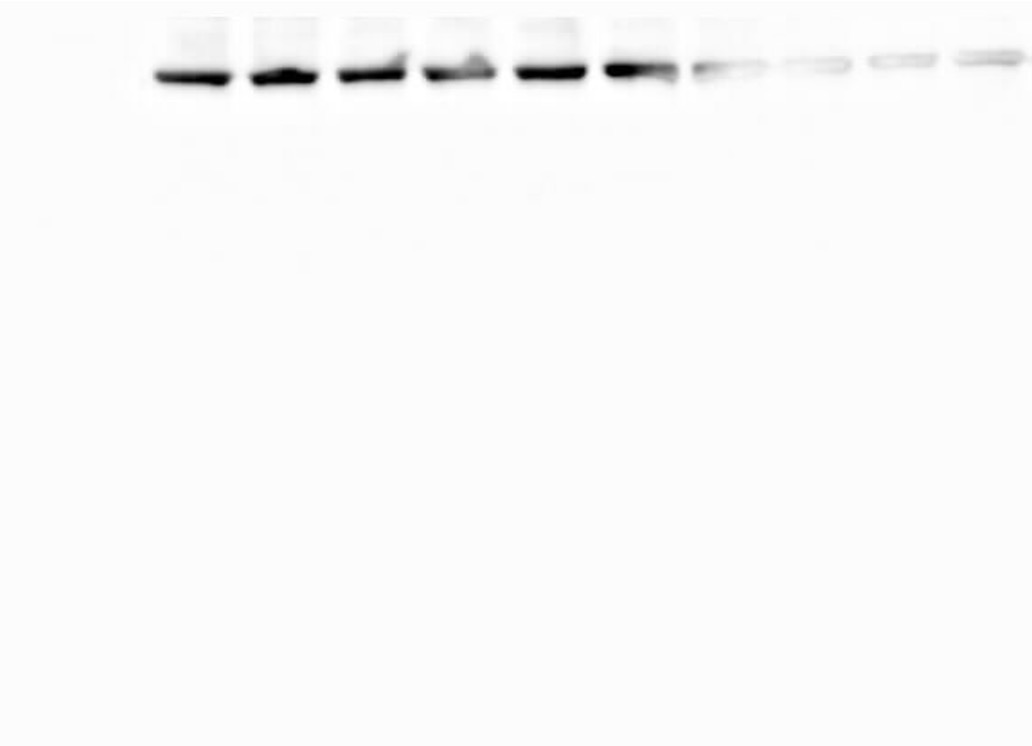

Supplement: Supplementary file 2 [file datasheet2.zip › JAK.jpg]

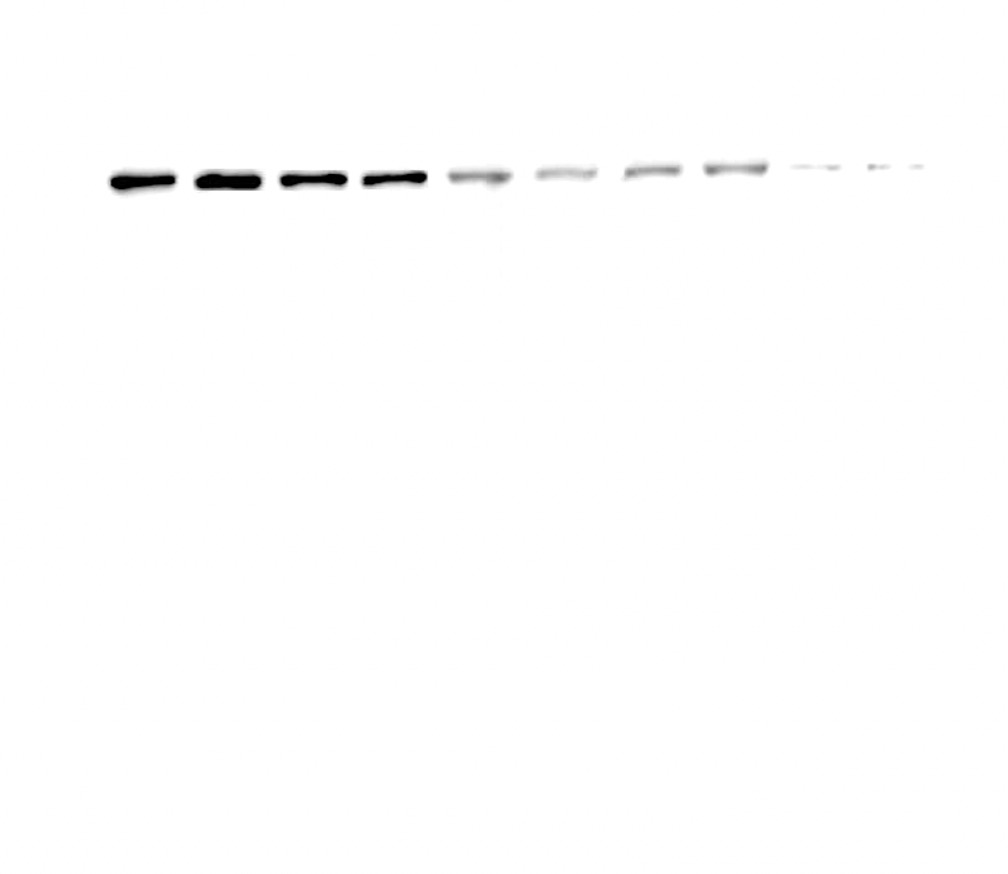

Supplement: Supplementary file 2 [file datasheet2.zip › pAkt.jpg]

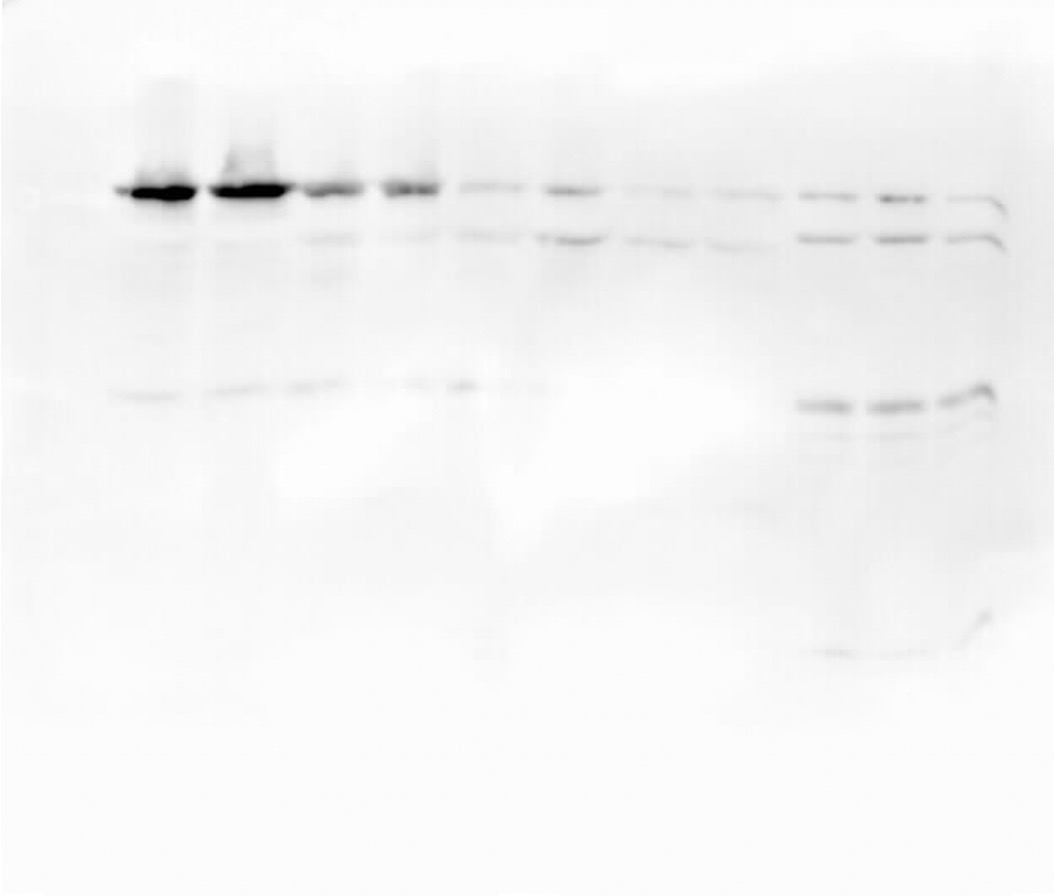

Supplement: Supplementary file 2 [file datasheet2.zip › PI3K.jpg]

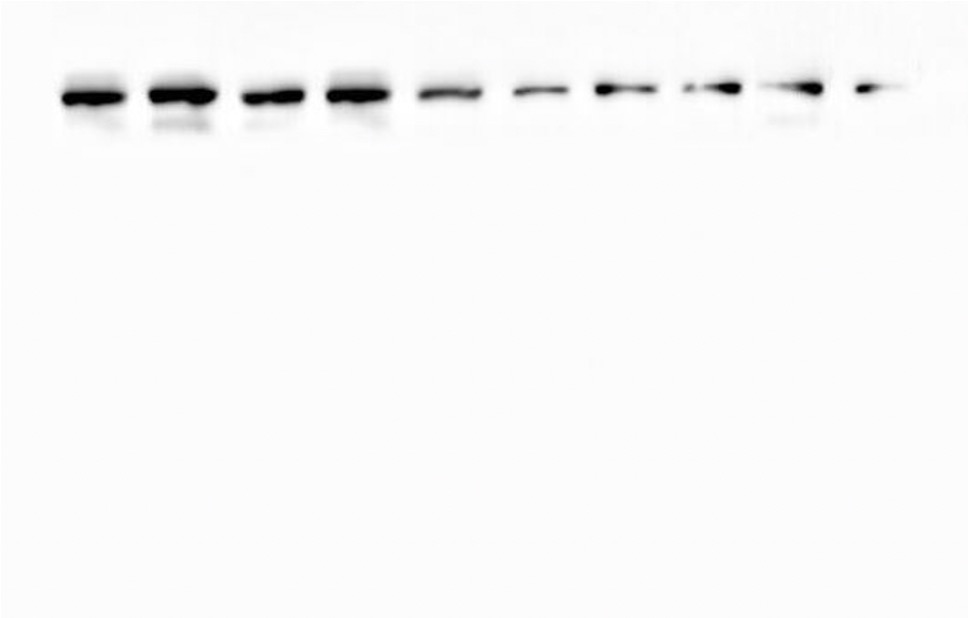

Supplement: Supplementary file 2 [file datasheet2.zip › pSTAT.jpg]

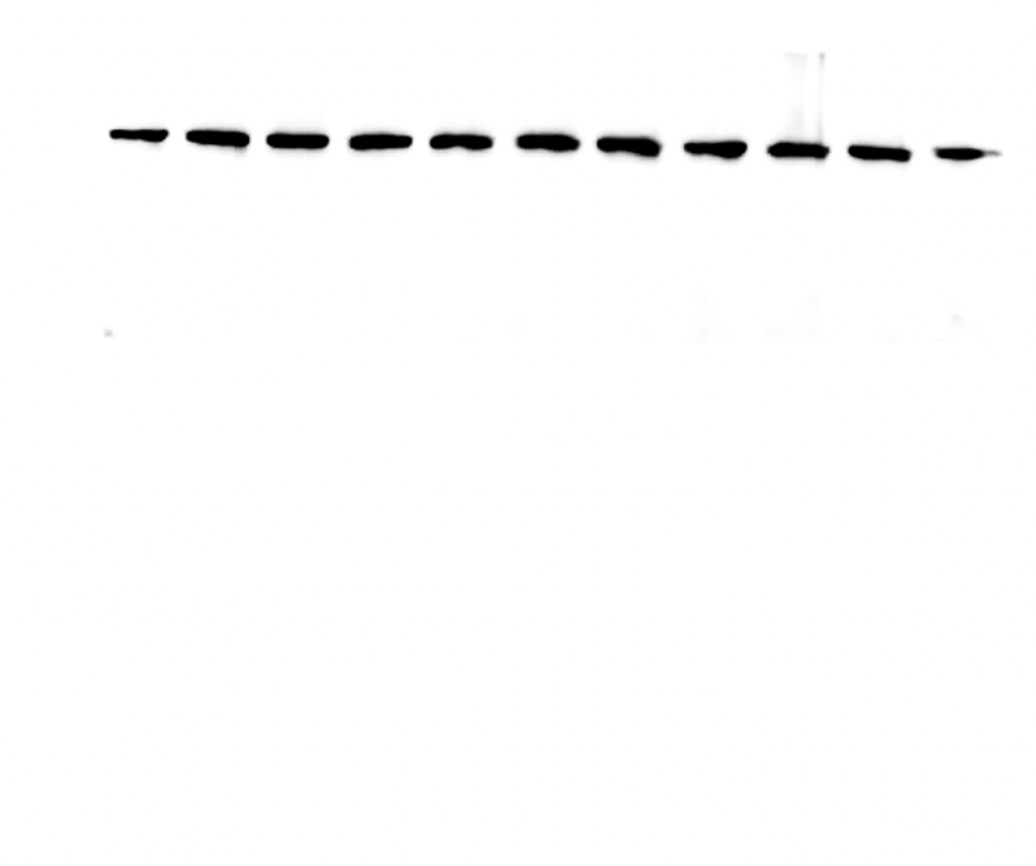

Supplement: Supplementary file 2 [file datasheet2.zip › STAT.jpg]
